# A digital twin model for evidence-based clinical decision support in multiple myeloma treatment

**DOI:** 10.3389/fdgth.2023.1324453

**Published:** 2023-12-20

**Authors:** Nora Grieb, Lukas Schmierer, Hyeon Ung Kim, Sarah Strobel, Christian Schulz, Tim Meschke, Anne Sophie Kubasch, Annamaria Brioli, Uwe Platzbecker, Thomas Neumuth, Maximilian Merz, Alexander Oeser

**Affiliations:** ^1^ Innovation Center Computer Assisted Surgery (ICCAS), University of Leipzig, Leipzig, Germany; ^2^ Department of Hematology, Hemostaseology, Cellular Therapy and Infectiology, University Hospital of Leipzig, Leipzig, Germany; ^3^ Clinic of Internal Medicine C, Hematology and Oncology, Stem Cell Transplantation and Palliative Care, Greifswald University Medicine, Greifswald, Germany

**Keywords:** digital twin, clinical decision support, multiple myeloma, knowledge graph, treatment outcome simulation, value-based healthcare

## Abstract

The treatment landscape for multiple myeloma (MM) has experienced substantial progress over the last decade. Despite the efficacy of new substances, patient responses tend to still be highly unpredictable. With increasing cognitive burden that is introduced through a complex and evolving treatment landscape, data-driven assistance tools are becoming more and more popular. Model-based approaches, such as digital twins (DT), enable simulation of probable responses to a set of input parameters based on retrospective observations. In the context of treatment decision-support, those mechanisms serve the goal to predict therapeutic outcomes to distinguish a favorable option from a potential failure. In the present work, we propose a similarity-based multiple myeloma digital twin (MMDT) that emphasizes explainability and interpretability in treatment outcome evaluation. We've conducted a requirement specification process using scientific literature from the medical and methodological domains to derive an architectural blueprint for the design and implementation of the MMDT. In a subsequent stage, we've implemented a four-layer concept where for each layer, we describe the utilized implementation procedure and interfaces to the surrounding DT environment. We further specify our solutions regarding the adoption of multi-line treatment strategies, the integration of external evidence and knowledge, as well as mechanisms to enable transparency in the data processing logic. Furthermore, we define an initial evaluation scenario in the context of patient characterization and treatment outcome simulation as an exemplary use case for our MMDT. Our derived MMDT instance is defined by 475 unique entities connected through 438 edges to form a MM knowledge graph. Using the MMRF CoMMpass real-world evidence database and a sample MM case, we processed a complete outcome assessment. The output shows a valid selection of potential treatment strategies for the integrated medical case and highlights the potential of the MMDT to be used for such applications. DT models face significant challenges in development, including availability of clinical data to algorithmically derive clinical decision support, as well as trustworthiness of the evaluated treatment options. We propose a collaborative approach that mitigates the regulatory and ethical concerns that are broadly discussed when automated decision-making tools are to be included into clinical routine.

## Introduction

### Personalized treatment approaches in multiple myeloma: the role of digital twin (DT) models

The treatment landscape for multiple myeloma (MM) has experienced substantial progress over the last decade. The development of multiple novel agents with different mechanism of actions, targeting both the myeloma cell and its microenvironment has significantly improved patients’ survival. Due to the availability of multiple drugs and drug combinations, therapy can be decided in an individualized approach, based on disease and patient's characteristics.

#### Complexity of the disease

The choice of treatment can vary significantly based on several factors, including whether the patient has recently been diagnosed, is a candidate for a stem-cell transplant, is dealing with relapsed/refractory multiple myeloma (RRMM), is considered to be at high risk due to the nature of his or her disease or has significant comorbidities. Each of these scenarios may require a different approach to treatment decision-making (1). Due to the fact that MM is still an uncurable disease, many patients receive five or more therapy lines in the course of their treatment journey (2). Taken together, various patient scenarios and the need for multiple lines of therapy make treatment decision in MM a multifaceted and challenging task.

#### Characterization depth

A good understanding of underlying disease mechanisms is crucial to the development of digital twins (DT). Furthermore, DT models heavily rely on substantial amounts of high-quality data. MM affects approximately 588,000 people worldwide each year, and accounts for 1% of all cancers and about 10% of all hematologic malignancies, making it the second most frequent hematological cancer (3, 4). Given its significance within these statistics, MM warrants comprehensive characterization and understanding, as well as extensive collections of patient data, medical records, molecular, and imaging data.

#### Treatment landscape

The treatment landscape of MM has evolved significantly over the last decades. Choosing the right medications for patients and determining the optimal timing and sequencing of these drugs is a complex task. It involves deciding which drugs to use, when to use them, and whether they should be administered in a specific order or combined together (1). Established and novel regimens are multifaceted and include alkylators, steroids, proteasome inhibitors (PIs), immunomodulatory drugs (IMiDs), histone deacetylase inhibitors (HDACi), monoclonal antibodies (mAbs), antibody-drug conjugates (ADCs), bispecific T-cell engagers (BiTEs), chimeric antigen-T-cell therapy (CAR-T), peptide-drug conjugates, selective inhibitors of nuclear export (SINEs) and small-molecule targeted therapy (5, 6). These agents are either used as single therapies or can be combined to doublet, triplet or quadruplet regimens and can be used as induction before autologous stem cell transplantation (ASCT), as continuous treatment or at the time of relapse (5). The diverse range of MM treatments presents a multitude of clinical scenarios that cannot be adequately addressed by a single standardized approach (7) and reflect the requirement for tailored approaches to meet the specific needs of individual patients.

### Applications and challenges of DTs in precision medicine

DTs are applied for different purposes in the field of healthcare, from the strategic organization of hospitals and processes to creating digital twins of patients to personalize medical care (8). In the following, we focus on patient-based applications, due to the fact that requirements and challenges for each high-level use-case vary significantly. In the overarching context of precision medicine, the goal of DT models is to enhance medical diagnostics, prognostics, treatment, and ultimately contribute to the overall improvement of a patient's well-being (9). Across different fields, a variety of models in every degree of maturity have been proposed. The field DTs have been studied broadest is cardiovascular disease, targeting issues from endovascular repair, hypertension and heart failure to electrophysiology. The models include data from EHRs, clinical and demographic data in general, imaging data, as well as electrocardiographic and tomographic databases. With methods from numerical analysis to deep neural networks and readiness levels from computational models and proof-of-concepts to semi-active DTs, the field is rapidly emerging (10). In the field of cancer, Filippo et al. have proposed a framework that integrates single-cell RNAseq data to set up digital metabolic twins (11). Additionally, roadmaps to DT development have been proposed by Angulo et al., who described a collaborative artificial intelligence (AI) and expert-based approach to personalized medicine in the field of lung cancer and Meraghani et al. proposed an DT approach for breast cancer detection using temperature data collected from portable devices (12, 13). Focusing on real-time applications, Zhang et al. developed a DT that serves as a monitoring system of elderly fall based on a vision sensor and a deep learning algorithm to model data on posture and behavior (14). Further DT models have been developed in the fields of multiple sclerosis (MS), Alzheimer's disease, nutrition, diabetes and orthopedics (15–22).

Since 2010, the development and publication of DTs in healthcare has risen exponentially. Nevertheless, the realization of clinical translation is still in its infancy, limiting the achievements of DTs in practice to date (23). Technical challenges in the development of DTs include the lack of individualized assessment and accuracy of validations, the scarcity of high-quality data, as well as the heterogeneity of data from multiple sources. In addition, ethical considerations include concerns of bias in AI models, as well as data privacy and safety regulations (23, 24).

### Methodological and technological prerequisites

Lakoff et al. argue that the term “experience” isn't synonymous with memory but instead the characterization of immediate, repetitive sensorimotor interactions with the surroundings, embodying a continual action (25). This repetition sustainably impacts the shape of functional neuron groups in the brain, leading to formation of patterns. In the context of clinical decision-making, this aligns with the procedure of recalling previous situations (derived from that pattern) that appear similar or familiar for the task at hand. However, the depth of such patterns is tied to an individual and thus, limited in its scale and granularity. Furthermore, while this behavior is considerably useful for common decision-making tasks, it becomes largely useless for rare or unforeseeable complex scenarios. One mechanism of overcoming uncertainty in such uncommon scenarios is the referral to observations made and reported by others, such as clinical trials or, more precisely, clinical case reports. In this way, learnings previously derived by others can be adapted to a present decision-making problem.

At scale, such aggregation and integration of observations enables the formation of a knowledge graph that represents a certain state of knowledge about a defined subject (26) using a mathematical graph as the underlying structure. Based on this representation, various functions can be subsequently implemented, such as the targeted search for evidence (27) or the ability to draw conclusions through the utilization of inference mechanisms (28). Especially recently, and due to significant methodological and technological achievements, such as in the field of machine learning (ML), new solutions and applications are continuously being established, which transfer valuable assistance into clinical routine (29, 30).

Referring to the first concept of the twin concept, introduced in the NASA Apollo program, replicas of the actual spacecraft were built to mirror its characteristics and functionalities and thus, enable simulation, training and scenario planning, i.e., in case of emergency (31). Following the definition proposed by Barricelli et al., we define DT as a computer-based model that simulates, emulates, mirrors and is therefore “twinning” the life of a physical entity to continuously predict future statuses (32). To specify further, Boulos et al. defined key concepts of human digital twins and stated that different DT *types* can be defined in the sense that DTs can be created for the whole body, a body function, organ or for a specific disorder. DT *instances* are copies of a DT belonging to the same individual for the use of *in silico* testing. DT *levels* define the degree of abstraction the model holds and a DT *thread* is the definition of the data pipeline over time (9). We transferred those concepts to create a dynamic model starting from time of diagnosis (*thread*) and based on clinical routine data (*level*) to simulate treatment (*instances*) in the field of MM (*type*). While in medicine, generating an exact copy of a patient or a disease is impossible to achieve, DTs can be based on specialized knowledge bases (or knowledge graphs) which, depending on their inherent quality and validity, are able to, i.e., simulate the behavior of cells and associated interactions to improve drug development (24). As such, the concept of a DT is embedded in a data model alongside an associated environment for interaction, distribution, and management.

The consideration of possible approaches to treating a patient is essentially based on a cognitive simulation of an expected outcome by the treating physician. In this process, considering the risk-benefit ratio is crucial, as invasive therapies bring both short- and long-term implications for the patients. Therefore, the potential of a DT in this context lies in the parallel evaluation of viable scenarios with respect to one or more outcome factors. Related approaches, analogous to clinical trials, primarily consider long-term endpoints such as overall survival (OS), progression-free survival (PFS) (33) or targeted aspects like the response of specific biomarkers (34). Stühler et al. developed a solution for outcome simulation of multiple endpoints in therapies against MS using Hierarchical Bayesian General Lineral Models, emphasizing factors of therapeutic effiectiveness, e.g., number of relapses and disability porgression, but not the associated invasiveness (35).

In this work, we describe a framework for multi-perspective evaluation of therapy success using a DT. We therefore aim to identify overarching concepts that help overcome the challenges involved in DT development and on that basis propose a model that levels with the digitization and automation readiness of clinical routines. The goal is to make sure that each suitable treatment for a patient is considered and the physician is supported in the comparison of individualized implications for each option. Our solution emphasizes the consideration of multiple parallel endpoints related to therapy-based implications as well as the adaptation of the underlying process to better suit chronic diseases with multiple lines of therapies. We have implemented our approach in the realm of MM patient stratification and therapeutic outcome evaluation, since the disease features a high degree of diagnostic granularity as well as a diverse range of applicable treatment options. Although the proposed solutions and findings refer exclusively to MM, they are intended to be generalizable for other diseases and clinical use-cases.

## Methods

In the first step, we performed a requirement analysis to define the overarching concepts that should be implemented in the MMDT. Therefore, we initiated a literature search, focused on the assessment of the state of the art regarding DT concepts and implementations. We further emphasized results targeting severe or chronic pathological conditions. The resulting methodological and architectural design was developed in alignment to our findings.

### Methodological and architectural concepts

**Figure 1:**
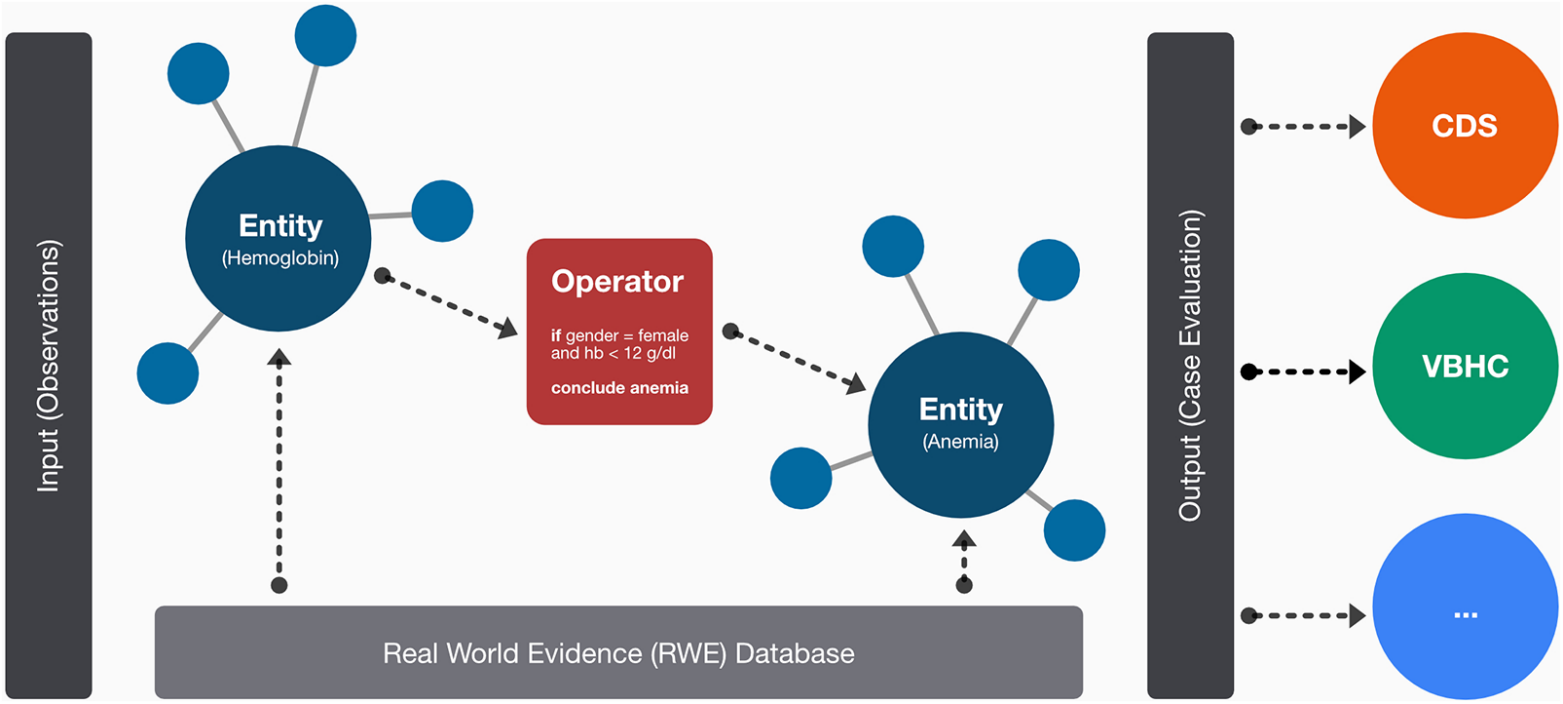
Schematic overview on the layer-based architecture of the MMDT involving dedicated entities for observed (i.e., hemoglobin) and calculated (i.e., anemia) parameters as well as the causal connections among them in a network-based structure. On the edges of the network, logical operators are used to represent the formal rulesets between connected entities. The resulting evaluation (e.g., for an integrated clinical case) can then be used for subsequent tasks or associated systems such as clinical decision-support systems.

The underlying architecture of our MMDT consists of four dedicated layers: (1) entity description, (2) entity network (3) formal logic and (4) real-world evidence (see Figure 1).

Within the entity description layer, we defined and formalized clinical parameters (referred to as entities) associated with the characterization of MM patients and the application as well as evaluation of therapeutic regimens. Patient characterization includes demographic data, medical history, clinical observations, laboratory reports, medical imaging reports, genetic data, as well as treatment history. We further specified each parameter with a dedicated value set, using predefined classes or ranges associated with corresponding units, i.e., for laboratory findings. Every entity was then translated into a Health Level 7 (HL7) FHIR resource, enabling sustainable interoperability and alignment with leading medical coding systems (LOINC and SNOMED-CT) to further ensure terminological consistency.

Additionally, a classification regarding the respective entity type was made. Possible states included “observed” if the associated value can be derived directly from a patient, treatment or process, i.e., a person's age or the name of a substance, or “calculated” if it is the result of a processing step based on one or multiple observations, i.e., the presence of hypercalcemia or the class of the International Scoring System (ISS) (36).

The individual entities were then put into context using a dedicated network layer. This entity network features the entity descriptions as nodes, as well as their causal relationships based on the evaluation of medical evidence, i.e., clinical practice guidelines (CPG), peer-reviewed scientific publications, medical textbooks. We implemented our entity network using the resource description framework (RDF) with the Terse RDF Triple Language (TURTLE) for rendering a graph-based layout for subsequent expert-based validation.

**Figure 2:**
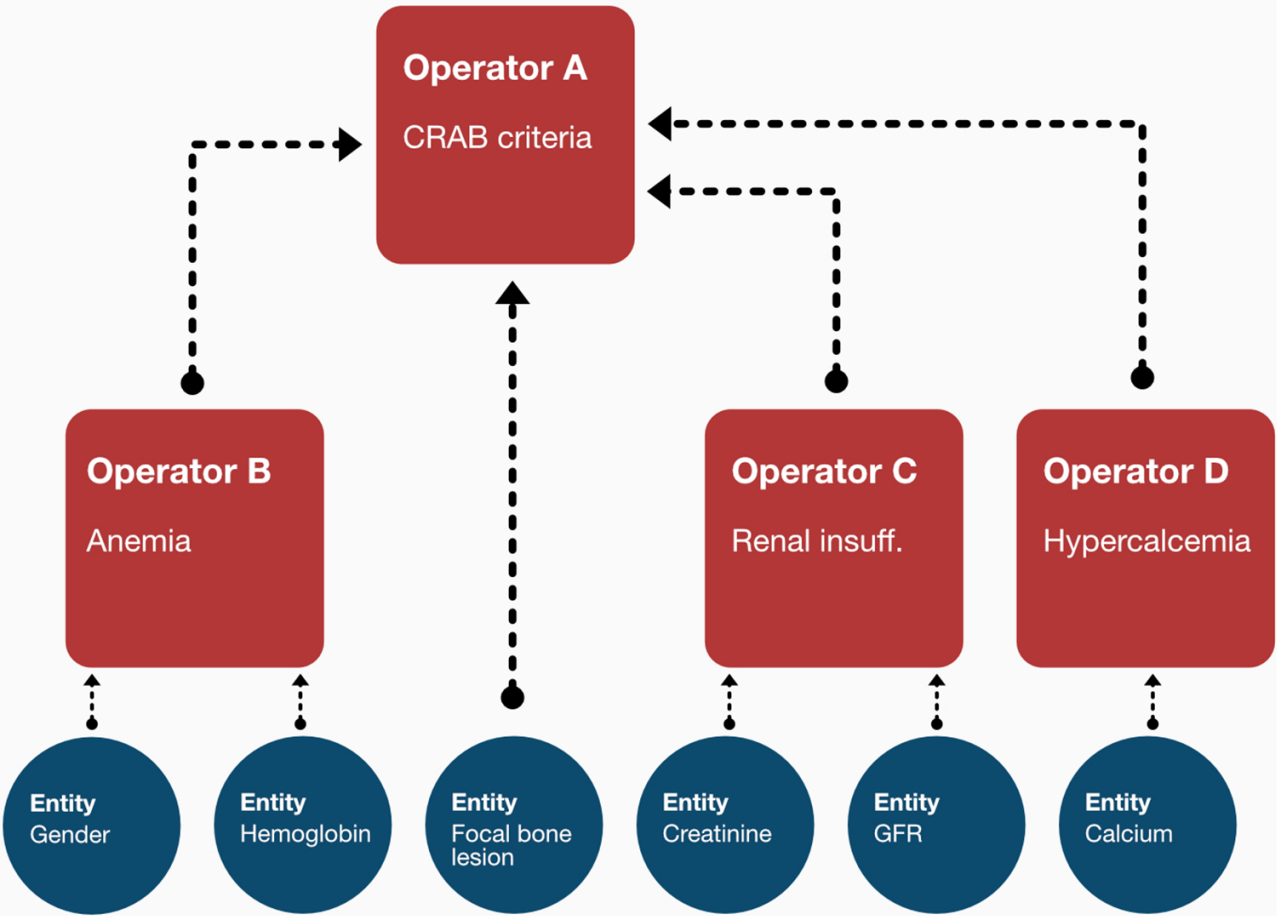
The recursive structure of the operators enables them to await dependent inputs from the output of other operators. In the shown sample case, the CRAB criteria, apart from the focal bone lesion observation, can only be assessed once the output of the anemia, renal insufficiency and hypercalcemia operators becomes available. The operator thus triggers the calculation of those entities first before any internal processing is made.

Following network implementation, we selected all entities of type “calculated” and implemented dedicated logic modules (referred to as operators) which contain a rule-set to derive the respective categorical (i.e., ISS) or numeric (i.e., Charlson-Comorbidity-Index) output (36, 37). While those operators are technically independent functions, they are designed to form a nested structure to align with the network environment (see Figure 2). Thus, they are implemented in a recursive fashion in which each operator is performing an input evaluation procedure to make sure that all required values (either based on observed data or the output of a previous operator) are available. If not, the corresponding precursive operators are triggered to provide the required results.

**Figure 3:**
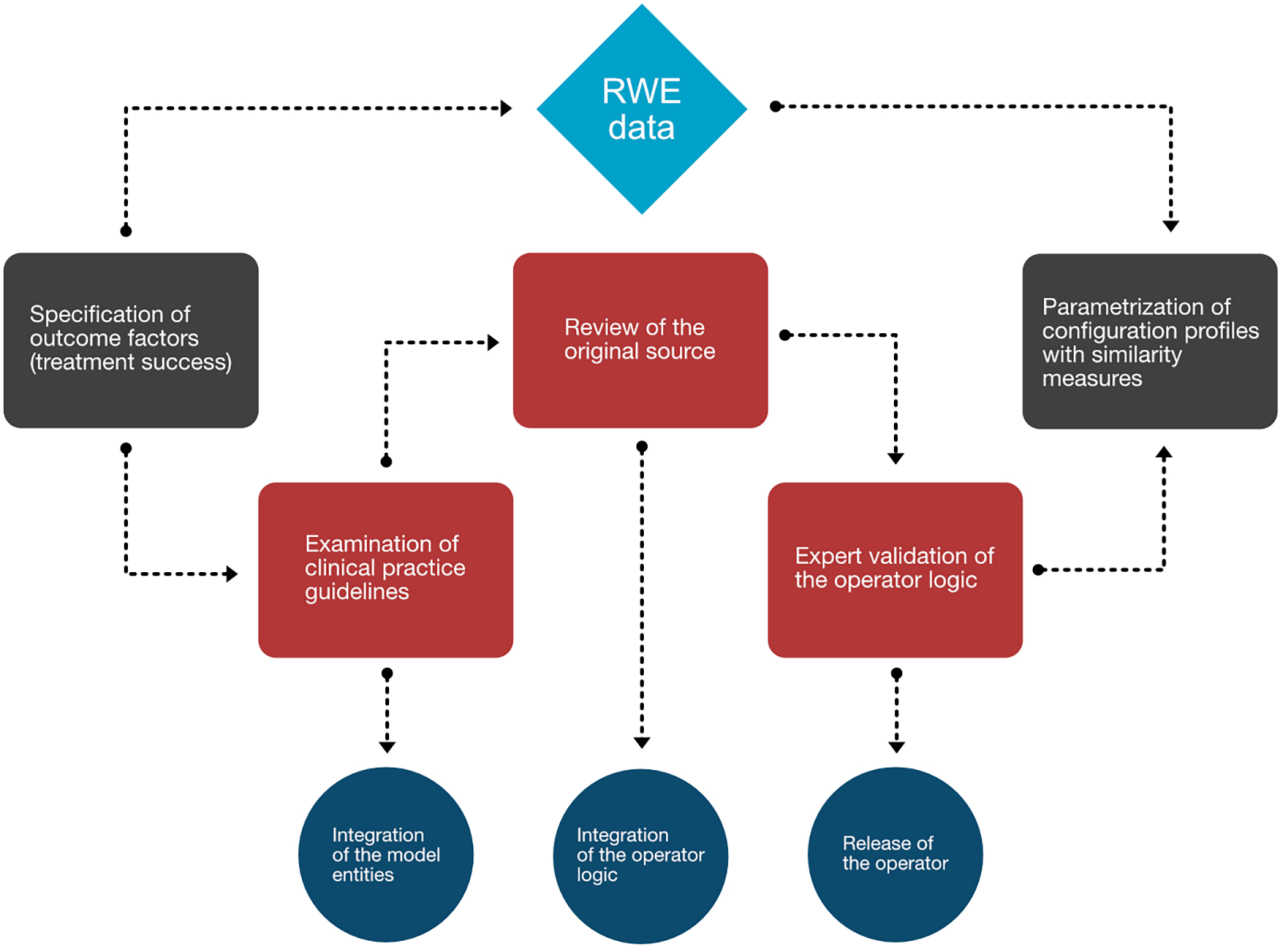
Workflow diagram mapping the process generating the MMDT knowledge base. Worksteps associated with manual evidence assessment are depicted in red, while translational steps are shown in dark blue. Associated events (gray) at the beginning and end of the process are directly tied to the RWE database (light blue).

We first determined the overarching picture about what aspects should be represented in the MMDT knowledge base, as shown in the workflow diagram (Figure 3). In the case of a chronic disease such as MM, an initial diagnosis of the disease is followed by iterative cycles of treatment and disease staging to observe therapeutic efficacy and the duration of positive treatment effects. We therefore first examined clinical factors associated with the evaluation of treatment success. Knowing that those outcome factors are dependent on a variable treatment entity, we then carefully examined the clinical practice guidelines (Onkopedia and Onkowissen) to derive the influential factors directly associated with the allocation of specific treatment strategies (i.e., patient condition) as well as the prerequisites of the MM diagnosis and staging (i.e., CRAB and SLiM criteria). Since those direct dependencies do not represent observable values but rather multifactorial scoring systems, we have consulted the original sources which defined and evaluated those entities in the first place. Following this procedure, we iteratively checked the dependencies of each entity in our network (i.e., the presence of anemia as a condition of CRAB) until only observable values are defined as the input of an operator. If this was the case, then no further logic needed to be introduced as all influential factors could be directly derived from clinical findings and reports.

While the deterministic nature of clinical scoring systems, i.e., performance, prognostic or evaluation scores, allowed for the use of rulesets for calculation and thus, patient stratification, this characteristic does not apply to the task of outcome simulation. Therefore, baseline information about the documented clinical outcomes of MM patients is required in order to build a fundamental knowledge base about the quantitative expressions between cause (patient characteristics) and effect (therapeutic outcome). In the case of DT-based applications offering clinical decision-support (CDS), the definition of characteristics for treatment efficacy can be conceived as endpoints which need to be closely aligned with the actual clinical process to ensure that the corresponding measurements or observations are part of routine diagnostics. Emphasizing the fact that the goal of MM therapy cannot be defined by a single metric, multiple endpoints in combination should cover the multi-perspective facets, e.g., reduction of disease burden as well as the risk for significant adverse events. Thus, we selected a set of meaningful endpoints to define what discriminates a good therapy option from a suboptimal one. The following selected and defined endpoints mirror a direct reaction to a specific line of treatment to assess context-dependent treatment success.

#### Remission

The 2006 International Myeloma Working Group (IMWG) criteria provide a standardized and internationally accepted way of comparing clinical treatment response (38). The criteria are commonly used in clinical trial design, to guide treatment decision making, to monitor treatment progression and to predict prognosis and are therefore utilized as an important endpoint in the DT model. In treatment strategies that include ASCT, this endpoint is assessed after induction therapy as well as after transplantation.

#### Time to next treatment

Time to next treatment (TTNT) is defined as the number of days from the start of an initial treatment to the start of the subsequent treatment. This provides an objective and quantifiable measure that follows the rationale, that the extension of the associated time period indicates that the treatment in question has effectively controlled the disease and slowed its progression. Many cancer treatments can be physically and emotionally cumbersome for patients, so in addition to being a clinical outcome measure, this endpoint acts as an additional proxy indicator for a patient's quality of life.

#### Adverse events

Considering the risk for significant adverse events, it is particularly important to assess the benefit-risk-ratio before any treatment application. The best responses in MM treatment are achieved with rather invasive treatments which are not suitable for frail patients. Consequently, a personalized evaluation of harmful side effects is crucial to guarantee treatment safety.

#### Quality of life

Addressing one important aspect of VBHC, quality of life measurements centering around a patient's well-being and focusing on individual perception of a disease's implications gain in significance. By incorporating the results of quality of life questionnaires, the patient's perspective can be included in clinical decision-making processes. Nowadays, those metrics are commonly part of clinical trial protocols but usually not of routine disease monitoring. In our MMDT, we incorporated the endpoint based on two validated questionnaires filled out by the patient: the EORTC QLQ-C30 and the EORTC QLQ MY20, that assess quality of life in cancer patients in general and particularly in MM, respectively (39, 40). In the scope of our implementation, the endpoint is defined as a comparison to patients in the same treatment stage based on retrospective data.

#### Early mortality

In treatment strategies that include ASCT, early mortality, defined as the death of a patient within 100 days after the intervention, is a clear indicator for personalized risk assessment. Thus, the respective endpoint contributes further to the benefit-risk-ratio assessment associated with MM therapy decision-making.

Alongside the specification of patient and outcome characteristics, attributes of the available treatment options (drugs or interventions such as ASCT or cell therapy) need to be considered as well. In utilizing the MMDT as an assistance tool in therapeutic decision-making, the major aim is to link specific treatments or the combination of multiple substances to the associated outcomes. To enable a consistent procedure of data processing within the MMDT, we defined each therapeutic option as a dedicated entity in the network and represented deterministic aspects between patient and treatment characteristics as operators. Thus, we were able to model potential conflicts and integrate those into an instantiated output, e.g., the exclusion of options from the list of therapeutic regimens when the patient is known to be refractory against a contained substance. Finally, we also included similar procedures to include relevant process information, i.e., observations derived from previous therapy lines. Thus, the MMDT is able to conclude that a specific option is eligible for continued patient application based on previously observed outcome criteria in combination with their temporal assignment.

### Implementation and initial evaluation process

As an architectural concept, the MMDT can be adapted to any kind of model, analysis, or clinical decision support system (CDSS), since it inherits a formal description about knowledge associated with MM and can be connected to a database with the associated RWE to allow for quantitative outcome assessments. In the work at hand, we describe an approach for patient characterization and therapy decision support as well as its practical implementation.

While the manifestation of the MMDT describes the entirety of conditions, an instantiation can be defined as a snapshot of one particular patient state in the context of a specific therapy line. The goal is to compare therapy options from different perspectives and assess their associated benefits and risks. To do so, treatment response and disease course for a specific patient is simulated through a twin cohort derived from an RWE database. The hypothesis is that the considered patient is expected to have a comparable reaction to proposed therapy regimens as the identified retrospective cases that were considered to be similar.

The definition of similarity-defining features as well as the corresponding thresholds that need to be respected are stored in configuration profiles which allow for intuitive expert-based validation. To account for incomplete data, we defined proxies for certain entities. As an example, the Eastern Cooperative Oncology Group (ECOG) performance status was used interchangeably with the Karnofsky index to assess the functional status of patients. More broadly, an overall fitness measure was introduced that, in addition, also considers age and comorbidities, scored through the Charlson-Comorbidity-Index (CCI).

To initially derive those constraints, we utilized CPGs and expert-based evaluations. Our approach relies on the identification of dedicated cohorts for each endpoint to account for known differences in outcome assessment based on a specific characteristic. For example, the similarity cohort for remission is based on cytogenetic high-risk assessment, while the cohort definition for quality of life assessment is not constrained on that characteristic. In contrast, fitness affects both response rates and quality of life and is therefore considered a defining factor in either one of the similarity cohorts (see Table 1). In this manner, the augmented patient profiles are matched with the RWE database according to the similarity constraints defined in the configuration profiles. As a result of the twin cohort identification for each endpoint, a set of medical cases with the chosen therapeutic approach and corresponding documented outcome is returned. Through an analysis of the observed outcome states, a probability distribution for each categorial endpoint is calculated and summarized to obtain a quantitative outcome assessment over a range of possible treatment strategies. The TTNT measure is calculated as the mean days in the respective similarity cohort. In addition, warnings and conflicts are calculated based on matching the patient's profile to information on guideline- and expert-based recommendations, known side effects and severe adverse events. For example, if a dose reduction for patients with renal insufficiency is recommended and a patient's lab results report creatinine levels above the reference range, a warning is inferred. Finally, we consolidate the CPG-based assessment to label the presented options as either CPG-compliant or off-label strategies.

**Table 1:** Definition of the similarity-defining features and application to the defined endpoints.

Endpoint specific twin cohort based on similarity measure						
Patient characteristics	Similarity measure	Remission	TTNT	QOL	AE	EM
Age	Fitness	yes	yes	yes	yes	yes
ECOG						
CCI						
t(4;14)	High-risk cytogenetics	yes	yes	no	no	yes
t(14;16)						
del(17p)						
gain(1q21)						
t(14;20)						
Albumin	ISS	yes	yes	no	no	yes
Beta-2-Microglubuline						

To demonstrate the implementation regarding patient stratification and therapeutic outcome simulation, we have constructed a representative RWE database based on first line treatments from the Multiple Myeloma Research Foundation (MMRF) CoMMpass dataset. CoMMpass is a prospective, longitudinal, observational study of newly diagnosed MM patients (41). To align with the specifications of the MMDT, we first identified and extracted all contained items associated with either patient-, disease-, process- or outcome-related characteristics and mapped them to the previously defined entity network. Subsequently, we have monitored the documented treatment landscape of the CoMMpass dataset and excluded cases that (1) featured (potentially outdated) therapeutic strategies which are not included in the current CPGs (42, 43) and (2) had incomplete outcome parameters according to our endpoint specification. We then generated a treatment evaluation scenario for an exemplary MM patient (see Table 2) to obtain first insights about the reasoning mechanics of the MMDT.

**Table 2:** Exemplary MM patient characteristics with derived the similarity measure as defined in Table 1.

Patient characteristics	Exemplary MM patient characteristics	Similarity measure	Examplary MM patient similarity measure
Age	55	Fitness	fit
ECOG	0		
CCI	2		
t(4;14)	no	High-risk cytogenetics	no
t(14;16)	no		
del(17p)	no		
gain(1q21)	no		
t(14;20)	no		
Albumin	3.1 g/dl	ISS	2
Beta-2-Microglubuline	3.3 mg/L		

## Results

### Initial requirements regarding the MMDT

#### The need for modularity

Significant advances have been made in biology, prognosis and therapy for MM patients in recent years. The growing number of treatment options, for both newly diagnosed and relapsed/refractory cases, has made the clinical landscape increasingly complex (7). With various new therapeutic options being studied, the landscape is expected to continuously evolve (6). Consequently, a MMDT must be constructed in a modular way, so that it can be easily updated and validated to stay up-to-date to the fast-changing clinical landscape.

#### Robust handling of data

Data gaps frequently pose a significant barrier when obtaining the best insights from clinical data (44). In addition, while randomized controlled trials (RCT) provide in-depth evidence for specific interventions, integrating real-world evidence (RWE) from a variety of sources is recognized as valuable for comprehensive healthcare decision-making (45). As a result, it is crucial for the MMDT to demonstrate robust capabilities in data management and handling. This includes the proficiency of handling incomplete data, incorporating RWE and RCTs, as well as datasets including evidence collected in a different treatment era.

#### Consideration of the patient perspective

Due to the chronic course of the disease, it is crucial to study how treatments affect MM patient's lives beyond extending survival times. Being exposed to different lines of therapy, patients can face a high symptom burden, increased cumulative toxicities and a poor health-related quality of life (46). Consequently, the MMDT should encompass multiple endpoints, including a focus on value-based healthcare (VBHC) by measuring outcomes from a patient perspective.

#### Traceability of reasoning mechanisms to ensure transparent results

To enhance trust in the system, it is recommended to minimize “black box” elements in data-driven applications and to guarantee that internal mechanisms of models are explained to users (47). Moreover, studies have indicated that users tend to overly depend on a system's recommendation, even when those are incorrect. It is suggested that providing explanations may help mitigate unwarranted trust and reliance in the system (48). As a result, we require the MMDT to prioritize transparency, explainability and interpretability and offer corresponding visual feedback to ensure optimal human result evaluation.

### Development results

In the entity description layer, our MMDT instance consists of 475 unique entities, either referring to patient, disease, treatment or process characteristics. The entity network features 438 connections among those entities to form the knowledge graph. While those specifications relate to a formal description of the MM context, only a subset is required for application in practical tasks such as clinical decision support or patient characterization. Referring to our initial evaluation scenario, targeting a treatment outcome simulation for MM patient in a first-line therapy, 23 observations and 17 logical operators are required to infer the respective medical scoring systems, while 10 factors and 3 operators are then applied to the subsequent patient stratification (see Table 2).

**Figure 4:**
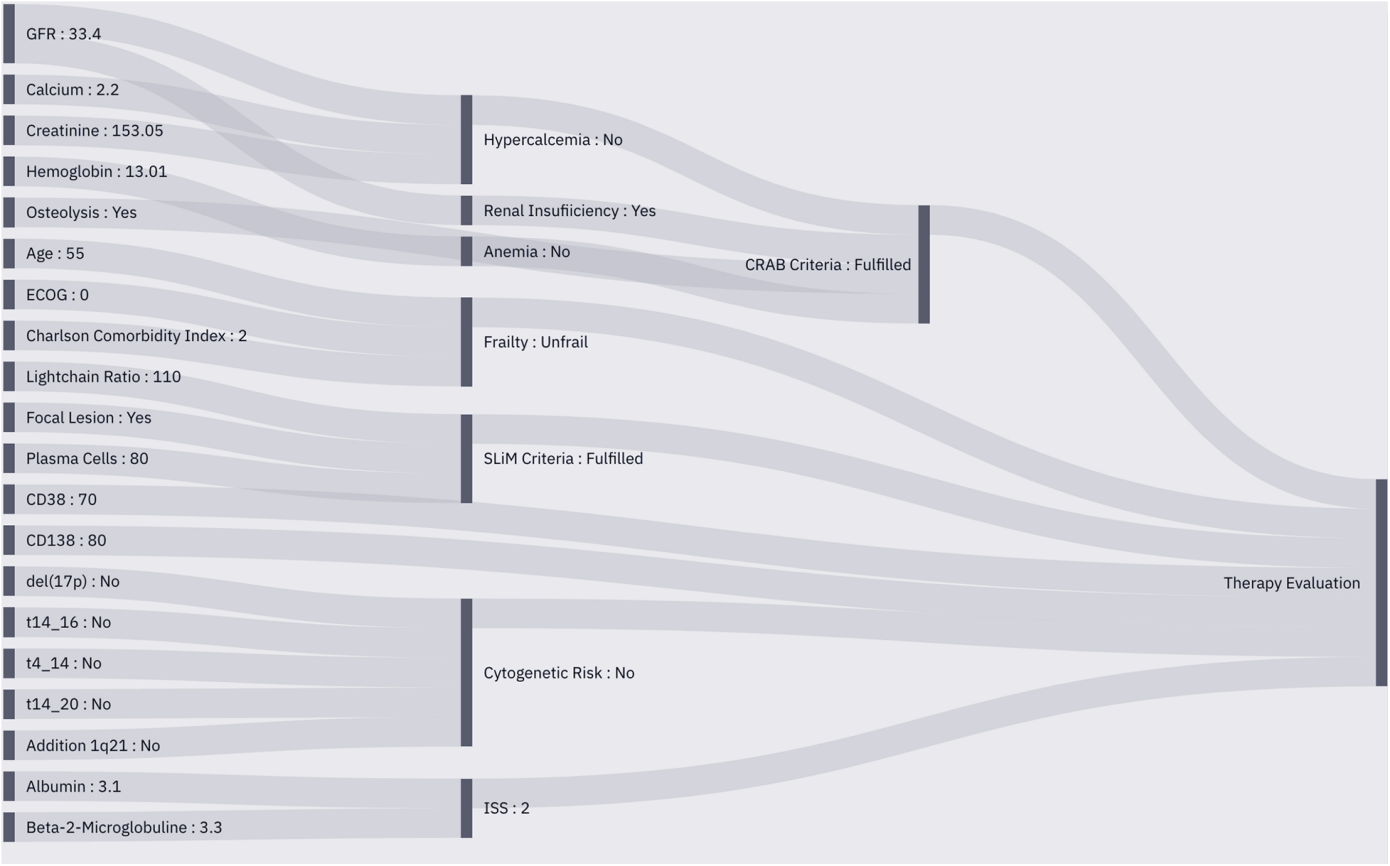
Sankey plot of the instantiated MMDT for patient characterization. Observed entities (left) are integrated into the operators to perform calculations based on their internal logic (middle), The pre-processed data is then utilized to perform therapeutic outcome simulation (right) using a similarity cohort from the RWE database.

Due to the internal connections, specified in the entity network and technically implemented in the operator modules, each instantiation of the MMDT automatically creates a procedural pathway that enables traceability of the performed information processing, i.e., output of operator A was inserted into the calculation of operator B which then led to the derivation of factor C as a preliminary or final result to the original request (see Figure 4).

Instantiation with a RWE database needs to align with specific requirements that are crucial to ensure valuable results. This includes the consideration of only validated evidence as well as the availability of obligatory values to perform the similarity search. While missing or sparse data is a constant issue in the medical domain, associated measures have been implemented in the MMDT to mitigate the issue. Considering the selection of similarity-defining metrics, fitness and high-risk cytogenetics each depend on multiple factors. In the case of fitness, both values, ECOG and CCI, are aimed to express the physiological status of a patient to characterize his or her ability to tolerate invasive treatment. In this scenario, the presence of one of those factors would be sufficient to allow for inferring a valid output. The same is true for high-risk cytogenetics where the detection of one out of five chromosomal aberrations would be enough to justify a high-risk label. However, in this particular scenario, the MMDT would not conclude the absence of the high-risk state based on incomplete data but only its presence.

**Figure 5:**
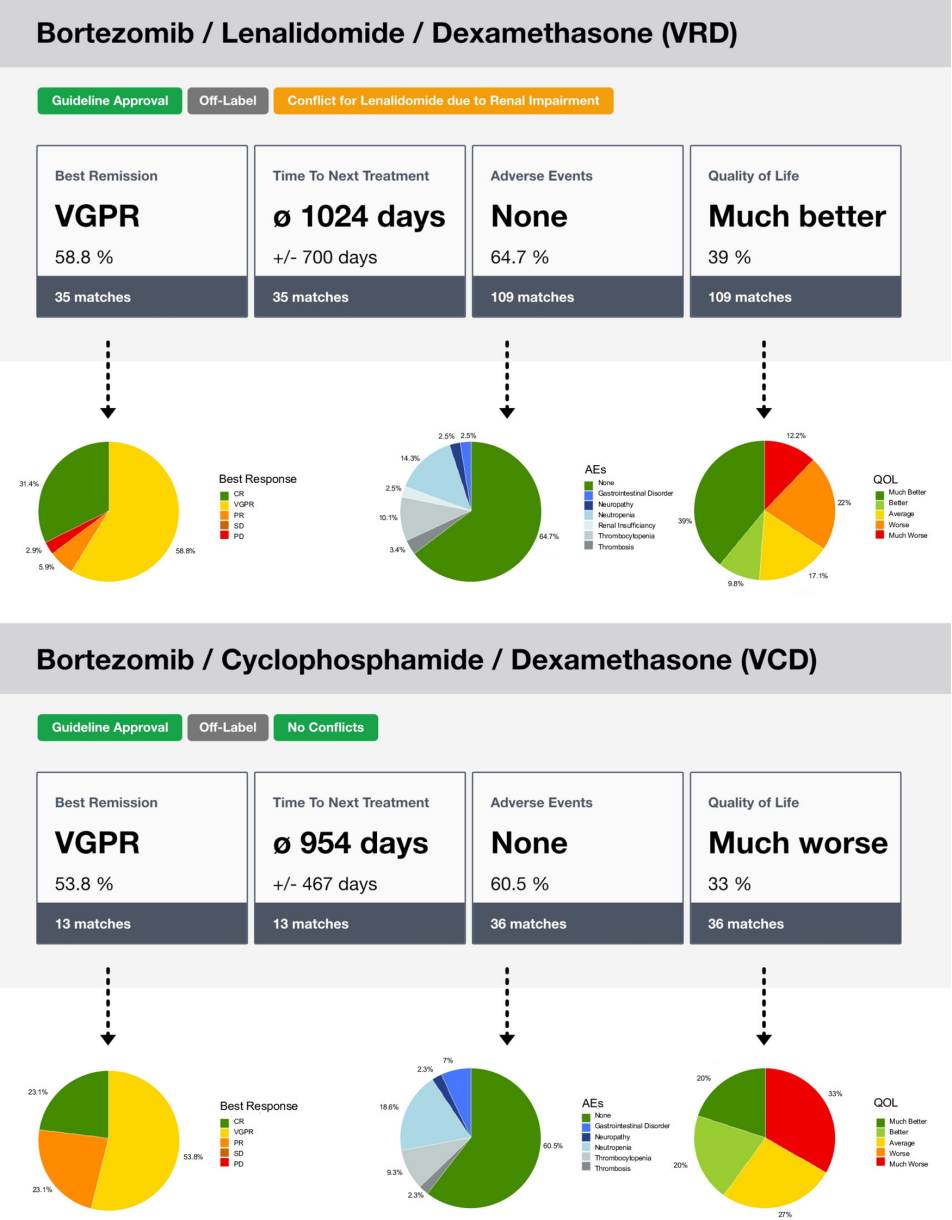
Illustration of the outcome simulation assessment through the MMDT. After the similarity cohort has been identified in the RWE database, the recorded endpoints are used to calculate the distribution of the observed outcome states. In the overview, the state with the highest percentage is displayed. In the associated details, the full distribution is presented. Furthermore, labels are used to show the state of approval, i.e., based on a CPG, or potential conflicts.

Regarding the instantiation of the MMDT for treatment outcome simulation with the connected CoMMpass RWE database and the exemplary MM patient case, we were able to process a complete outcome assessment based on the previously introduced procedures. The output provides evidence on two common MM treatment regimens: Bortezomib, Lenalidomide, Dexamethasone (VRD) and Bortezomib, Cyclophosphamide, Dexamethasone (VCD). Both were assessed in combination with ASCT due to the unimpaired overall condition of the test patient (see Table 2). Further, the MMDT found evidence about subsequent maintenance therapies with either Bortezomib, Lenalidomide or Ixazomib. Using the input provided, the MMDT was able to correctly infer the patient characteristics based on the integrated operator logic, leading to a valid selection of therapeutic options according to the corresponding CPG. Further, a representative distribution of potential outcomes has been derived (see Figure 5), showing improved results with the VRD regimen, which also represents a more current approach in first-line MM treatment. Emphasizing on accountability, the described MMDT does not flag one therapy option as ideal but leaves the final decision to the physician. Thus, he or she can interpret the merit of each option by individually assessing the influences each outcome assessment might have on the final treatment decision.

## Discussion

With the proposed MMDT model we presented an approach closely aligned to the clinical practice, transitioning the concept to a practical application. We recognize that further research and work is needed to close the gap between the complexity of the disease and the MMDT as described. As discussed subsequently, several aspects can be additionally considered in future implementations.

Due to our layer-based approach, we've achieved a high level of modularization, further refined within the intra-layer architecture, i.e., through the implementation of nested operators. This was identified as a key characteristic in the DT development: modularity makes sure that changes in one module do not affect other modules (32). In the ever-evolving clinical domain this feature is especially important. In this way, we were also able to ensure sustainable maintenance of the underlying knowledge base, as new entities and their corresponding logic can be easily integrated and connected with the existing network through the mere specification of input and output dependencies. For example, if new evidence on a treatment is introduced, it is highly important that the DT features this update timely and low in effort. Especially in treatment facilities that do not have the capacities to always stay up-to-date with the latest research in a rapidly evolving entity as MM, this directly improves patient care. This approach further enabled effective validation, as both automated testing procedures as well as expert-based evaluation can be handled on a single-unit level. However, besides the advantages targeting modularization and transparency, the procedure also introduces significant overhead as a single change to the overarching knowledge base triggers a modification of several components within the architecture. In addition, a DT system requires high levels of maintenance not required in a highly specialized CDSS system (49). Thus, a higher potential for errors is introduced. The need for synchronization between all layers is especially relevant at the interface between the actual MMDT instance and the RWE database. Since the entities need to exactly align with the documented keys and defined values, each violation would exclude the whole RWE case from consideration in the outcome simulation process.

Another obstacle lies in the consolidation of large high-quality clinical datasets. Clinical data is often fragmented across institutions and systems. Privacy regulations limit sharing of patient data between healthcare providers further complicating the consolidation of large datasets. Apart from regulatory obstacles, healthcare providers follow different guidelines and protocols in diagnostics and treatment. These variations occur on an international level, but even on national, regional and institutional level. Depending on local clinical practice guidelines, healthcare financing, regulatory bodies, institutional policies, center size and resources as well as level of innovation, the local standards for treating similar cases vary significantly. Moreover, clinical data is often documented in an unstructured or semi-structured manner that leads to the need for heuristic approaches in the data harmonization process. In the DT model at hand, interoperability standards were introduced to overcome challenges in clinical data heterogeneity within and across institutions. We were further able to enhance data interoperability through the implementation of the HL7 FHIR standard for entity specification, nevertheless notable limitations, such as generalization have been reported (50). In the process of mapping the MMDT entities into FHIR profiles, we found that the pre-defined high-maturity resources suitable to address the requirements of a highly specialized domain like MM have to be complemented with numerous custom profiles to fully specify all entities. The described approach overcomes several exclusion reasons of certain types of data such as clinical trials or datasets that feature outdated therapeutic strategies. Even though the choice of treatment might not have been deliberate (e.g., randomized trials) or optimal, the signal about a therapy's efficacy for an individual is still valuable and interpretable. In general, the more data can be included in the model, the higher the number of patient characteristic permutations and consequently, the higher the level of precision in the evaluation. In the future, DT models will greatly benefit from the introduction of electronic health records (EHR), enabling improved data sharing and interoperability. The definition of a DT can feature the requirement of data exchange in real time between the physical entity (patient) and the virtual copy (51). A possibility of real-time updating of a patient's profile would improve the accuracy of therapy decision support models. Additionally, automatic data synchronization would provide further incentive for health care providers to use a system that does not require manual data entry.

An optimal DT model would include all variables that are relevant to MM pathogenesis. Especially with the progress of multi-omics techniques, this leads to models of immense proportions and the corresponding computational challenges. To date, there is a wide gap between this complexity of MM and routine health care, making the integration and exploitation of vast amounts of data an ongoing challenge (52). Nevertheless, examining the gene expression patterns is widely regarded as a highly reliable method for investigating various aspects of a cell, including its identity, current condition, function and response (53). Genomic analyses of tissue samples from myeloma patients have shown that MM represents a spectrum of hematological entities with extensive tumor heterogeneity. Multiple layers of this complexity have already been studied in multi-omics settings, including inter- and intra-patient diversity, disease state, relapse status, treatment response and spatial heterogeneity in bone disease (54–60). Results comprehensively highlight the need for profiling clonal heterogeneity and molecular processes associated with MM at each development stage (61). Creating a holistic DT model for MM should therefore integrate multi-omics data in the future to address the disease's intricate inherent complexity. Another potential data source presents digital phenotyping, which is defined as “moment-by-moment quantification of the individual-level human phenotype in-situ using data from smartphones and other personal digital devices” (62). This automated and objective data collection about lifestyle, physiological state and environment can then be combined and support other data sources to reach the goal of personalized medicine (62, 63).

Besides technical challenges, there are ethical aspects that need to be considered. It is crucial to be aware of the possible introduction of bias reflected in multiple aspects when designing a DT model. First, bias can be introduced if the data used to create the basis for the model is not a diverse representation of patients in terms of age, gender, race, ethnicity, and other demographic factors. Furthermore, potential bias can be added when collecting clinical data from a limited number of medical centers or within specific healthcare systems. In addition, algorithms simply learn from persisting and uncorrected bias in the healthcare system, reflecting human biases. For example, if an algorithm suggests a group of patients are more likely to choose aggressive care at the end of life, this might be based on historical care patterns that might not apply to an individual patient's or family's treatment preferences (64). In most models, the fit of the majority population is more significant to the overall error than the fit to the minority population (65). This poses a challenge, even if the data underlying the model perfectly fits the averaged metrics of the disease's demographic. As an example, MM is mainly characterized by advanced age and has a higher prevalence in men than in women (42), resulting in a very low representation of young females in a representative dataset. This is true for a wide variety of demographic and clinical factors and must be kept in mind when constructing a twin model based on real-world data. A general approach to address these issues is that enhancing transparency greatly facilitates bias detection and improves comprehension of a program's erroneous decisions (66, 67).

In recent years, many tools that replicate medical specialists' performance have been developed and can provide guidance in all fields of clinical decision making. Nevertheless, stakeholders have broadly stated that CDSS should not replace but merely support doctors in decision making. Clinicians' issues around trust and trustworthiness were identified as control and liability. It was emphasized that it is crucial that clinicians have the final responsibility to make decisions regarding diagnosis and treatment. Evidently, an automatization in the proposed setting would not be feasible, consequently the proposed CDSS leaves the physician 100% in control. In consequence, this design choice is not optional in the sense that only a system trusted in by physicians can make a positive impact on a patient's treatment in the first place. The second major issue refers to medical errors and liability in a sense that if a doctor's decision diverged from the CDSS recommendation (68). Our model addresses this strongly by not designating a single therapy as the best choice but instead grants the physician the authority to make a decision based on all information available. In summary, our collaborative approach mitigates the regulatory and ethical concerns that are broadly discussed when automated decision-making tools are to be included into clinical routine. To date, the MMDT is implemented as an isolated service to be utilized on request, but next development steps include the integration into existing clinical infrastructures.

To pilot test the model, several key aspects need to be considered to ensure its effectiveness, safety and reliability. In this sense, it is important to highlight the different objectives of the MMDT system and the associated RWE database. While the major goal of the system is to perform valid and useful processing of the incoming data, the inherent value of the database is based on its ability to reflect an objective representation of the patient and treatment landscape regarding MM. Only in this way, the selected similarity cohorts can provide clear signaling towards therapeutic outcome assessment. Thus, an evaluation procedure targeting the value of the data-driven assumptions is only possible once the utilized RWE is able to align with those requirements. However, certain features of the MMDT can be tested independently of the quality of the dataset, this includes user feedback on visualization and presentation of results as well as interoperability with different types of clinical information systems. All aspects of the CDSS, as well as triggered conflicts and warnings need to be tested for correctness. Future users should further evaluate the usefulness of the system in terms of saving time in clinical routines and the potential of improving treatment courses and patient care in general.

## Data Availability

The original contributions presented in the study are included in the article/Supplementary Material, further inquiries can be directed to the corresponding author.
